# Clinical outcomes of maxillary sinus augmentation in atrophied posterior maxilla based on residual bone height

**DOI:** 10.1186/s40902-025-00484-7

**Published:** 2025-09-29

**Authors:** Donghyun Lee, Jinlee Kim, Hyejin Kim, Seunggon Jung, Min-Suk Kook, Hong-Ju Park, Jaeyoung Ryu

**Affiliations:** 1 Department of Oral and Maxillofacial Surgery, School of Dentistry, Chonnam National University, Gwangju, Republic of Korea; 2 Department of Oral and Maxillofacial Surgery, Chonnam National University Hospital, Gwangju, Republic of Korea

**Keywords:** Maxillary sinus augmentation, Residual bone height, Implant success, Bone graft resorption, Clinical outcome

## Abstract

**Background:**

The posterior maxilla is often the site of early tooth loss, frequently requiring sinus augmentation to compensate for vertical bone deficiency. However, the influence of residual bone height (RBH) on clinical outcomes remains unclear.

**Materials and methods:**

This retrospective study evaluated 42 patients who underwent maxillary sinus augmentation using either the lateral or transcrestal approach. Patients were divided into two groups: the severely atrophied group (RBH < 3 mm) and the moderately atrophied group (RBH 3–5 mm). Clinical outcomes assessed included marginal bone loss (MBL), graft resorption, complications, and implant survival.

**Results:**

Clinical outcomes were comparable regardless of RBH. Even in severely atrophied maxillae with RBH < 3 mm, favorable outcomes were achieved using appropriate surgical techniques.

**Conclusion:**

RBH should inform surgical planning but should not be considered a strict prognostic indicator.

## Background

The maxillary posterior region, particularly the area of molars, is frequently the first to experience tooth loss, often due to periodontal disease [1, 2]. Following tooth extraction, implant-supported prostheses are frequently necessary to restore both function and esthetics. However, implant placement in this region is often challenging due to anatomical limitations, including alveolar ridge resorption and maxillary sinus pneumatization [3]. Furthermore, the posterior maxilla typically exhibits lower bone density compared to other regions, necessitating meticulous planning to ensure sufficient primary stability for implants [4].

Sinus floor augmentation has become a widely accepted and reliable technique for addressing vertical bone deficiencies in the posterior maxilla. The lateral window technique and the transcrestal approach are most employed. The transcrestal approach, first described by Summers in 1994, offers advantages such as reduced invasiveness, shorter surgical time, and lower postoperative morbidity [5]. However, it presents limitations including restricted visibility and technical difficulty in verifying membrane integrity during the procedure [6]. To overcome these challenges, various modified transcrestal techniques—utilizing hydraulic pressure, specialized osteotomes, and osseodensification drills—have been developed to enhance the safety and predictability of sinus elevation [7]. Conversely, the lateral approach, introduced by Boyne and James in 1980, allows for direct visualization of the sinus membrane and offers greater control during graft placement [8]. This makes it particularly suitable for cases involving severely atrophic ridges or anatomical variations like sinus septa [9]. Nevertheless, it is associated with higher risks of membrane perforation and surgical morbidity [10].

Historically, the selection between transcrestal and lateral approaches has been based on the residual bone height (RBH), with the lateral approach traditionally favored when RBH is less than 5 mm and the transcrestal approach recommended for RBH exceeding 5 mm [11]. However, recent evidence suggests that with appropriate technique, successful outcomes can be achieved using the transcrestal approach even in cases where RBH is less than 5 mm [12]. The high success and survival rates of implants placed after maxillary sinus augmentation have been well-documented in numerous systematic reviews. For instance, it has been reported that implants placed after sinus augmentation demonstrated a 3-year survival rate exceeding 90%, indicating the high predictability of this procedure [13]. More recently, a meta-analysis suggested that non-grafted sinus lift procedures could also yield successful outcomes under specific conditions, sparking discussions on the diversity and evolution of the technique [14]. While numerous studies have investigated clinical outcomes following sinus floor augmentation, controversies persist regarding the precise influence of RBH on implant success and complication rates. Therefore, this study aims to comprehensively compare clinical outcomes, including implant survival, complication rates, marginal bone loss (MBL), and graft resorption, between cases presenting with RBH less than 3 mm and those with RBH between 3 and 5 mm.

## Patients and methods

The study was conducted with the approval of the Institutional Review Board of Chonnam National University Dental Hospital (IRB No. CNUDH-2025-017). All procedures were conducted in accordance with the ethical standards of the committees responsible for human experimentation, and with the Declaration of Helsinki of 1975 as revised in 2008. This retrospective study analyzed the clinical and radiographic outcomes of 42 patients who underwent maxillary sinus augmentation between July 2016 and November 2022. The study included adult patients (aged 18 years or older) who required maxillary sinus augmentation for implant placement. To ensure the independence of observations, a single implant site from each patient was included in the analysis, resulting in a total of 42 independent data points. Eligibility was determined based on the availability of preoperative cone-beam computed tomography (CBCT, CS9600, Carestream Dental LLC, Atlanta, GA, USA) scans for accurate measurement of RBH and cases where implant-supported prostheses were successfully delivered. Patients with systemic conditions contraindicating surgery (e.g., severe cardiovascular disease, immunosuppressive conditions, or recent history of chemotherapy or radiotherapy to the head and neck region) were excluded. Additionally, cases with insufficient clinical or radiographic records or noncompliance with follow-up protocols were excluded to ensure data reliability. The implant systems used in this study were the Osstem TSIII SA® and USII® (Osstem Implant, Busan, Korea), and the AnyOne® and BLUEDIAMOND IMPLANT® (MegaGen Implants, Daegu, Korea). All fixtures featured a sandblasted, large-grit, acid-etched (SLA) surface. For all sinus augmentation procedures, a freeze-dried bone allograft (FDBA) with a particle size of 250–1000 µm (OraGRAFT®, LifeNet Health®, Virginia Beach, VA) was used as the graft material.

Clinical data were meticulously collected from electronic medical records, encompassing patient demographics, implant-related details, timeline variables, health factors, and outcome measures. Patient demographics include age and gender. Surgical, implant-related and anatomical details comprised residual bone height, sinus and membrane morphology, surgical approach (lateral or crestal), type and brand of implant used, implant length and diameter, and timing of implant placement (immediate or delayed). Timeline variables included the stage, loading, and radiographic follow-up periods. Health-related factors such as smoking status, diabetes, and use of drugs for osteoporosis were also recorded. The primary outcome measures were MBL, graft resorption, and complications such as sinus membrane perforation or implant failure.

**Fig. 1:**
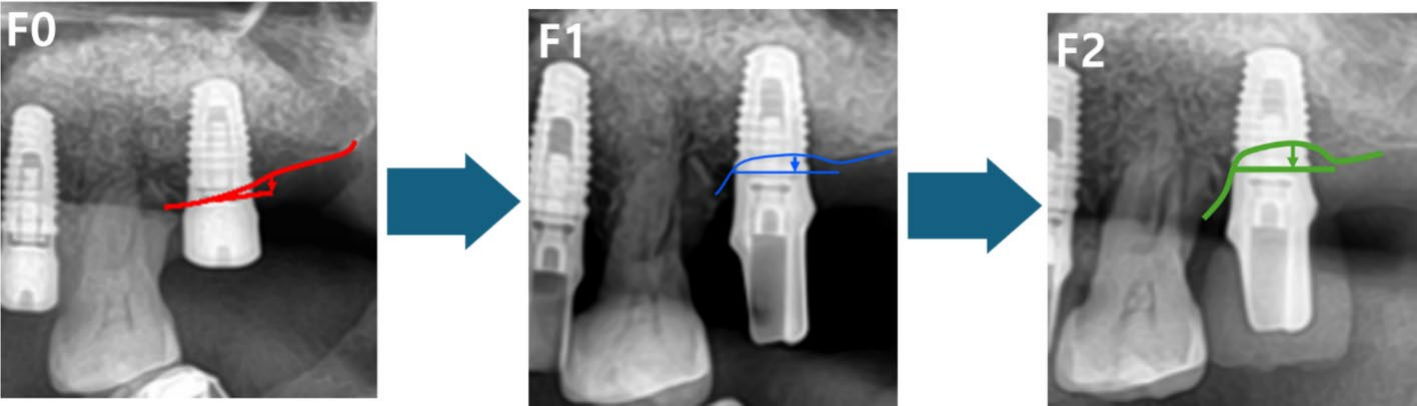
Diagram illustrating the measurement of marginal bone loss (MBL) on panoramic radiographs. MBL was calculated as the distance (in mm) from the crest to the top of the fixture at three points: F0 (immediately after implant installation), F1 (prosthesis delivery date), and F2 (final radiographic date). Marginal bone loss A (MblA) was calculated as F1 – F0, and marginal bone loss B (MblB) as F2 – F0

**Fig. 2:**
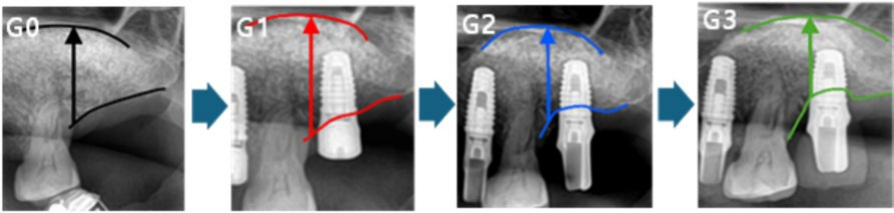
Diagram illustrating the measurement of graft resorption on panoramic radiographs. Graft height was measured from the crest to the top of the graft material at four time points: G0 (immediately after bone graft), G1 (immediately after implant installation), G2 (prosthesis delivery), and G3 (final radiographic date). Graft resorption A (GrA) was calculated as G1 – G0, graft resorption B (GrB) as G2 – G0, and graft resorption C (GrC) as G3 – G0

Radiographic evaluations were performed to assess MBL and graft resorption using panoramic radiographs and CBCT scans. MBL was quantified as the change in bone height from the crest of the ridge to the top of the implant fixture at three distinct time points: F0 (immediately after implant installation), F1 (at prosthesis delivery), and F2 (at the final follow-up visit) (Fig. 1). Graft resorption was evaluated by measuring changes in graft height at four predefined reference points: G0 (immediately after bone grafting), G1 (at implant installation), G2 (at prosthesis delivery), and G3 (at the final follow-up visit) (Fig. 2). These measurements were obtained using digital calipers within the INFINITT PACS M6 system (INFINITT Healthcare, Seoul, Korea). Residual bone height (RBH) was determined on preoperative CBCT scans as the vertical distance from the crest of the ridge to the sinus floor. Patients were categorized into two groups based on their RBH: severely atrophied maxillae (RBH less than 3 mm, *n* = 27) and moderately atrophied maxillae (RBH 3–5 mm, *n* = 15). This classification facilitated a comparative analysis of clinical and radiographic outcomes between the groups.

Statistical analyses were performed using RStudio software (Version 4.3.1; RStudio Inc., Boston, MA, USA). Continuous variables, such as age, MBL, and graft resorption, were expressed as mean ± standard deviation and analyzed using independent t-tests or Mann–Whitney U tests, depending on data distribution. Categorical variables, including smoking status and other health factors, were analyzed using the Chi-squared test or Fisher’s exact test. To assess relationships between variables, multiple regression analysis was conducted. The multiple regression model used to evaluate the effect of RBH on outcomes was adjusted for a range of covariates, including demographic, surgical, implant-related, anatomical, and health factors. Statistical significance was set at *p* < 0.05.

## Results

### Patient demographics

A total of 42 patients were enrolled in this study, with 27 in the severely atrophied maxilla group (RBH < 3 mm) and 15 in the moderately atrophied maxilla group (RBH 3–5 mm). The mean age was 59.93 ± 14.03 years in the severely atrophied group and 64.33 ± 12.34 years in the moderately atrophied group, with no significant difference. Gender distribution was also comparable between the groups, showing no statistically significant difference (Table 1).

**Table 1:** Comparison of basic patient demographic variables between severely and moderately atrophied maxillae

		Severely atrophied group (RBH < 3 mm, *n* = 27)	Moderately atrophied group (RBH 3-5 mm, *n* = 15)	*P*-value^*^
Age (Mean ± SD)		59.93 ± 14.03	64.33 ± 12.34	0.3154^a^
Sex (n)	Male	16 (59.26%)	11 (73.33%)	0.5646^b^
	Female	11 (40.74%)	4 (26.67%)	

^a^Independent t-test

^b^Chi-squared test

^*^p < 0.05

### Surgical, implant-related, and anatomical variables

All patients in the severely atrophied group underwent sinus augmentation using the lateral approach, whereas 40% of the moderately atrophied group received augmentation via the crestal approach. This difference in approach was statistically significant (*p* = 0.0009). Other key details showed no statistically significant differences between the two groups (Table 2).

**Table 2:** Comparison of key surgical, implant-related, and anatomical variables between severely and moderately atrophied maxillae

		Severely atrophied group (RBH < 3 mm, *n* = 27)	Moderately atrophied group (RBH 3-5 mm, *n* = 15)	*P*-value*
Approach (n)	Lateral	27	9	**0.0009**^b*^
	Crestal	0	6	
Placement Timing (n)	Simultaneous	24	15	0.5414^b^
	Staged	3	0	
Implant brand (n)	Osstem	20	11	1.000^a^
	MegaGen	7	4	
Implant type (n)	External	23	12	0.6858^b^
	Internal	4	3	
Implant diameter (n)	Wide (> = 4.8 mm)	7	5	0.8345^b^
	Regular (4.2 ~ 4.7 mm)	14	8	
	Narrow (< = 4.1 mm)	6	2	
Implant length (n)	> = 8 mm	27	15	-
	7 mm	0	0	
	< = 6 mm	0	0	
Sinus floor shape (n)	Flat	10	7	0.311^b^
	Sloped	10	7	
	Septa	7	1	
Sinus width (n)	Wide (> 12 mm)	13	11	0.2095^a^
	Narrow (< = 12 mm)	14	4	
Membrane morphology (n)	Normal (Thickness < 2 mm)	10	5	0.8253^b^
	Thickened (> = 2 mm)	10	4	
	Dome	2	2	
	Thickened with dome	5	4	

^a^Chi-squared test

^b^Fisher’s exact test

^*^p < 0.05

### Timeline variables

The time to prosthesis delivery was significantly longer in the severely atrophied group (9.15 ± 4.63 months) compared to the moderately atrophied group (5.60 ± 1.06 months) (*p* = 0.0059). However, no significant differences were found in the radiographic follow-up period or the staged period between the groups (Table 3).

**Table 3:** Comparison of timeline variables between severely and moderately atrophied maxillae

	Severely atrophied group (RBH < 3 mm, *n* = 27)		Moderately atrophied group (RBH 3-5 mm, *n* = 15)		*P*-value*
	Mean ± SD	Median (Range)	Mean ± SD	Median (Range)	
The time to prosthesis delivery (month)	9.15 ± 4.63	8.0 (4 –26)	5.60 ± 1.06	5.0 (4 – 7)	0.0059^a*^
Radiographic follow-up period (month)	18.78 ± 19.98	13.0 (0 – 75)	16.40 ± 12.39	16.0 (0 – 53)	0.6788^a^
Stage period (day)	16.44 ± 48.06	0 (0 – 180)	0.00 ± 0.00	0 (0 – 0)	0.1813^b^

^a^Independent t-test

^b^Mann Whitney U test

^*^p < 0.05

### Health factors

A statistically significant difference was observed in smoking status between the two groups, with a higher proportion of smokers in the moderately atrophied group (RBH 3–5 mm) (*p* = 0.0343). However, there were no significant differences between the groups in terms of diabetes or use of osteoporosis medication (Table 4).

**Table 4:** Comparison of health factor variables between severely and moderately atrophied maxillae

		Severely atrophied group (RBH < 3 mm, *n* = 27)	Moderately atrophied group (RBH 3-5 mm, *n* = 15)	*P*-value*	O/R (95% CI)
Smoking (n)	No	23 (85.19%)	8 (53.33%)	0.0343^*^	4.8128 (0.94–29.06)
	Yes	4 (14.81%)	7 (46.67%)		
Diabetes (n)	No	23 (85.19%)	10 (66.67%)	0.2415	2.7971 (0.49–17.42)
	Yes	4 (14.81%)	5 (33.33%)		
Osteoporosis medication (n)	No	25 (92.59%)	12 (80.00%)	0.3295	3.0339 (0.31–40.94)
	Yes	2 (7.41%)	3 (20.00%)		

Fisher’s exact test

^*^p < 0.05

### Complications

No statistically significant differences were observed. All implants were successfully placed, and no cases of implant failure were reported during the observation period (Table 5).

**Table 5:** Comparison of complications between severely and moderately atrophied maxillae

		Severely atrophied group (RBH < 3 mm, *n* = 27)	Moderately atrophied group (RBH 3-5 mm, *n* = 15)	*P*-value*	O/R (95% CI)
Membrane perforation (n)	No	26	12	0.1223^a^	6.9150 (0.45–353.18)
	Yes	1	3		
Implant failure (n)	No	27	15	-	-
	Yes	0	0		

^a^Fisher’s exact test

^*^p < 0.05

### Marginal bone loss and graft resorption

**Fig. 3:**
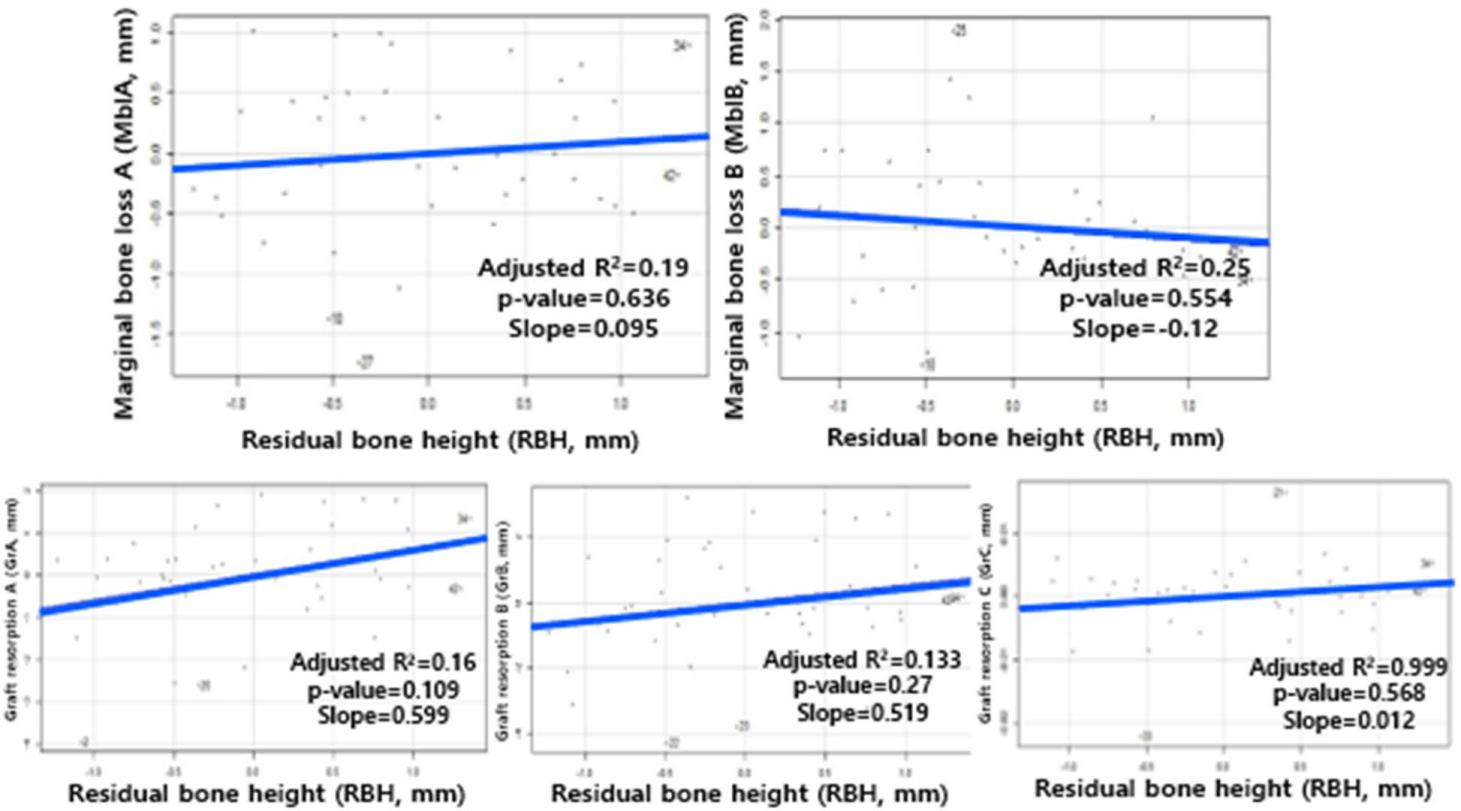
Partial regression plots for the effect of RBH on MBL and graft resorption variables. Multiple regression analysis indicated that RBH was not a statistically significant predictor of graft resorption or MBL after multivariable adjustment

Overall, both groups showed gradual marginal bone loss and reduction in graft height over time (Table 6). To assess the independent effect of residual bone height (RBH) and other clinical variables on these outcomes, a multiple regression analysis was performed in the fully adjusted model. In a multiple regression model adjusted for multiple variables, smoking was identified as a statistically significant predictor of marginal bone loss at the time of prosthesis delivery (MblA; *p* = 0.024). However, the analysis indicated that neither RBH nor any of the tested anatomical variables were found to be a statistically significant predictor of MBL or graft resorption (*p* > 0.05). The partial regression plots visually confirm the non-significant effect of RBH after accounting for these confounders (Fig. 3).

**Table 6:** Descriptive statistics of marginal bone loss and graft resorption between the two RBH groups

	Severely atrophied group (RBH < 3 mm, *n* = 27)		Moderately atrophied group (RBH 3-5 mm, *n* = 15)	
	Mean ± SD	Median (Range)	Mean ± SD	Median (Range)
MblA	−0.99 ± 1.03	−0.70 (−3.60 – 0.00)	−0.73 ± 0.83	−0.50 (−2.50 – 0.00)
MblB	−0.95 ± 1.22	−0.50 (−4.20 – 0.00)	−0.85 ± 0.85	−0.60 (−2.80 – 0.00)
GrA	−0.09 ± 0.29	0.00 (−0.90 – 0.00)	0.00 ± 0.00	0.00 (0.00 – 0.00)
GrB	−1.37 ± 2.03	−0.70 (−6.50 – 0.00)	−0.91 ± 1.25	−0.20 (−3.70 – 0.00)
GrC	−2.02 ± 2.87	−0.80 (−9.60 – 0.00)	−1.17 ± 1.95	−0.20 (−5.10 – 0.00)

## Discussion

This retrospective study evaluated the clinical and radiographic outcomes of maxillary sinus augmentation in patients with severely atrophied maxillae (RBH < 3 mm) compared to those with moderately atrophied maxillae (RBH 3–5 mm). Favorable outcomes were achieved in both groups. The distribution of surgical approaches in this study was consistent with conventional guidelines: all patients with RBH less than 3 mm underwent lateral sinus augmentation, while a portion of those with RBH between 3 and 5 mm received the transcrestal approach [11, 12]. This aligns with the widely accepted recommendation that the lateral approach is preferred for RBH 4 mm or less, as initially proposed by Summers and further supported by subsequent studies [5, 15]. However, several recent studies have demonstrated that the transcrestal approach can also yield successful outcomes in patients with RBH 4 mm or less, provided that proper technique and appropriate case selection are employed [12, 16].

In the present study, no significant differences were found between the two RBH groups regarding MBL or graft resorption. Consistent with prior research, implant success and peri-implant bone changes do not appear to be solely dependent on RBH when other surgical and prosthetic parameters are optimally managed [12, 17]. Furthermore, graft resorption did not differ significantly based on the degree of atrophy, supporting existing evidence that effective bone gain can be achieved irrespective of RBH if appropriate grafting and membrane elevation techniques are utilized [18].

The mean waiting period before implant prosthesis treatment was significantly longer in the severely atrophied group, likely attributable to the necessity of extended healing in cases of greater bone deficiency [19]. All implants in both groups achieved 100% survival, reinforcing the notion that delayed loading can enhance outcomes in severely atrophic cases.

An important finding of our multiple regression analysis was the identification of smoking as a significant predictor for early marginal bone loss (MblA). This is consistent with a large body of evidence that establishes smoking as a major risk factor for peri-implant complications [20]. The detrimental effects of smoking on peri-implant tissues are well-documented and are generally attributed to nicotine-induced vasoconstriction, which impairs blood supply and nutrient delivery to the healing site. This can compromise the initial stages of osseointegration and lead to greater early bone remodeling. Our results underscore the clinical importance of identifying smoking status during treatment planning and thoroughly counseling patients on the increased risk for early implant-related complications.

The timing of implant placement also differed between the groups, although not to a statistically significant degree (*p* = 0.0621). The severely atrophied group, which exclusively underwent the lateral approach, had a higher proportion of staged placements (92.6%) compared to the moderately atrophied group (66.7%). While our study did not find a significant difference in MBL outcomes, the extended healing period associated with staged procedures may have contributed to the favorable bone stability observed in the severely atrophied group [21].

Correlation and regression analyses revealed no significant associations between RBH and either MBL or graft resorption. This finding is consistent with previous studies reporting that residual bone height was not an independent predictor of bone gain or implant success after controlling for confounding variables [22]. This suggests that while RBH remains an important consideration for surgical planning, its role as a sole determinant of long-term clinical success may be limited when appropriate treatment protocols are diligently followed.

Of course, sinus augmentation is not the only solution for rehabilitating the atrophic posterior maxilla. Short implants (e.g., ≤ 6 mm) have emerged as an attractive, less invasive alternative. A recent retrospective study demonstrated high long-term survival rates for 4-mm short implants over a mean follow-up of 8 years [23]. This approach offers the advantages of reduced surgical time, lower morbidity, and greater patient acceptance. However, factors such as the crown-to-implant ratio and biomechanical load distribution must be carefully considered [24, 25]. Therefore, the choice of treatment should be individualized based on a comprehensive assessment of residual bone quality and quantity, occlusal factors, and patient-specific needs and preferences.

This study has several limitations. First, the retrospective design inherently introduces potential selection bias, and certain clinical parameters, such as implant stability quotient or long-term functional outcomes, were not available for assessment. Second, the sample size, particularly in the moderately atrophied group (RBH 3–5 mm, *n* = 15), was relatively small. This may have reduced the statistical power for some comparisons, and thus, the findings should be interpreted with caution. Although relationship between clinical outcomes and anatomical variables such as sinus and membrane morphology is also important point, this investigation was not performed in this study due to the lack of the numbers of the subjects. Future prospective studies with larger cohorts are needed to validate our results. Third, a formal assessment of intra-examiner reliability, such as an ICC, was not performed, which is a limitation of this study. Furthermore, the measurements were not conducted by a blinded, independent examiner, which could introduce potential measurement bias. However, all measurements were conducted by a single examiner to maintain consistency. Fourth, the radiographic follow-up period was highly variable among patients and relatively short in some cases, which may not be sufficient to capture long-term bone remodeling. Fifth, while this study included two different implant brands, this potential source of heterogeneity was statistically controlled for by including the implant brand as a covariate in our multiple regression models. Lastly, radiographic evaluation included both panoramic images and CBCT scans, and reliance on two-dimensional imaging for some measurements may limit precision compared to a comprehensive volumetric CBCT analysis [26]. Additionally, while this study quantified the overall vertical change in graft height, it did not include an analysis of the resorption pattern (e.g., coronal versus apical remodeling), which could provide further insights into the long-term biological behavior of the graft and remains an important area for future investigation.

## Conclusion

Within the limitations of this retrospective study, both lateral and transcrestal sinus augmentation techniques yielded comparable clinical outcomes in patients with residual bone height (RBH) less than 5 mm. No significant differences were found between the < 3 mm and 3–5 mm RBH groups in terms of marginal bone loss, graft resorption, or complication rates. However, the multiple regression analysis did identify smoking as a significant predictor for early marginal bone loss. Despite the more frequent use of the lateral approach in severely atrophic cases, implant survival was consistently high. These findings suggest that, with appropriate technique and case selection, successful sinus augmentation is achievable even in highly atrophic posterior maxillae. Therefore, although RBH should inform surgical planning, it should not be viewed as a definitive predictor of clinical prognosis.

## Data Availability

No datasets were generated or analysed during the current study.

## Abbreviations

RBH: Residual bone height
MBL: Marginal bone loss
